# Artificial Intelligence-Based Organ Delineation for Radiation Treatment Planning of Prostate Cancer on Computed Tomography

**DOI:** 10.1016/j.adro.2023.101383

**Published:** 2023-10-14

**Authors:** Eirini Polymeri, Åse A. Johnsson, Olof Enqvist, Johannes Ulén, Niclas Pettersson, Fredrik Nordström, Jon Kindblom, Elin Trägårdh, Lars Edenbrandt, Henrik Kjölhede

**Affiliations:** a Department of Radiology, Institute of Clinical Sciences, Sahlgrenska Academy, University of Gothenburg, Gothenburg, Sweden; b Department of Radiology, Region Västra Götaland, Sahlgrenska University Hospital, Gothenburg, Sweden; c Department of Electrical Engineering, Region Västra Götaland, Chalmers University of Technology, Gothenburg, Sweden; d Eigenvision AB, Malmö, Sweden; e Department of Medical Radiation Sciences, Institute of Clinical Sciences, Sahlgrenska Academy, University of Gothenburg, Gothenburg, Sweden; f Department of Medical Physics and Biomedical Engineering, Region Västra Götaland, Sahlgrenska University Hospital, Gothenburg, Sweden; g Department of Oncology, Region Västra Götaland, Sahlgrenska University Hospital, Gothenburg, Sweden; h Department of Clinical Physiology and Nuclear Medicine, Lund University and Skåne University Hospital, Malmö, Sweden; i Department of Molecular and Clinical Medicine, Institute of Medicine, Sahlgrenska Academy, University of Gothenburg, Gothenburg, Sweden; j Department of Clinical Physiology, Region Västra Götaland, Sahlgrenska University Hospital, Gothenburg, Sweden; k Department of Urology, Institute of Clinical Sciences, Sahlgrenska Academy, University of Gothenburg, Gothenburg, Sweden; l Department of Urology, Region Västra Götaland, Sahlgrenska University Hospital, Gothenburg, Sweden

## Abstract

**Purpose:**

Meticulous manual delineations of the prostate and the surrounding organs at risk are necessary for prostate cancer radiation therapy to avoid side effects to the latter. This process is time consuming and hampered by inter- and intraobserver variability, all of which could be alleviated by artificial intelligence (AI). This study aimed to evaluate the performance of AI compared with manual organ delineations on computed tomography (CT) scans for radiation treatment planning.

**Methods and Materials:**

Manual delineations of the prostate, urinary bladder, and rectum of 1530 patients with prostate cancer who received curative radiation therapy from 2006 to 2018 were included. Approximately 50% of those CT scans were used as a training set, 25% as a validation set, and 25% as a test set. Patients with hip prostheses were excluded because of metal artifacts. After training and fine-tuning with the validation set, automated delineations of the prostate and organs at risk were obtained for the test set. Sørensen-Dice similarity coefficient, mean surface distance, and Hausdorff distance were used to evaluate the agreement between the manual and automated delineations.

**Results:**

The median Sørensen-Dice similarity coefficient between the manual and AI delineations was 0.82, 0.95, and 0.88 for the prostate, urinary bladder, and rectum, respectively. The median mean surface distance and Hausdorff distance were 1.7 and 9.2 mm for the prostate, 0.7 and 6.7 mm for the urinary bladder, and 1.1 and 13.5 mm for the rectum, respectively.

**Conclusions:**

Automated CT-based organ delineation for prostate cancer radiation treatment planning is feasible and shows good agreement with manually performed contouring.

## Introduction

Accurate organ contouring for radiation treatment planning (RTP) of various forms of cancer, including prostate cancer (PCa), is challenging.^1^^,^^2^ Despite the availability of international RTP guidelines, practices may vary across radiation oncologists, treatment centers, and countries,^3^, ^4^, ^5^ due in part to inconsistent manual organ delineations.^1^ It is well-documented that manual organ contouring is resource- and time-consuming and subject to interobserver variability.^1^^,^^4^^,^^6^^,^^7^ Additionally, it has been shown that protocol deviations during RTP can result in inefficient treatment outcomes^3^^,^^5^^,^^8^ and decreased overall survival.^9^

Automated organ contouring could help alleviate these problems, as it would require less manual input and time and could provide a more standardized template for RTP of both the target organ and the surrounding organs at risk (OAR). Artificial intelligence (AI)-based algorithms have emerged as useful tools in oncological imaging and radiation oncology for localization, staging, and particularly for delineation of various forms of cancer, including malignancies in the pelvic region.^10^, ^11^, ^12^ It has been shown that AI can perform organ contouring faster and with greater accuracy than conventional methods.^13^, ^14^, ^15^

The application of AI for RTP of prostatic malignancies has primarily been focused on magnetic resonance imaging (MRI).^14^^,^^15^ This is mainly because of the higher soft-tissue contrast of MRI compared with computed tomography (CT), which enables easier differentiation of the tumor from the surrounding normal tissues, including OAR.^2^^,^^16^, ^17^, ^18^, ^19^ On the other hand, the use of CT for RTP could be more cost-effective and more globally accessible than MRI. To date, studies on automated delineations of the prostate and OAR on CT have been limited. We previously developed an AI-based algorithm for automated delineation of the prostate in high-risk patients with PCa using CT images of positron emission tomography/CT examinations. The findings showed a Sørensen-Dice similarity coefficient (DSC) of 0.78 to 0.79, which was comparable to the interobserver variability of manual delineations by participating radiologists in the study.^20^

The aim of the current study was to further develop our existing AI algorithm to achieve fully automated organ contouring of the prostate and the surrounding OAR using CT scans obtained before RTP. The accuracy of the AI algorithm was evaluated by comparing the output of the algorithm with manual delineations performed by radiation oncologists.

## Methods and Materials

### Patients and study design

The single-center patient cohort included 1530 consecutive patients with PCa from the local oncological department at Sahlgrenska University Hospital, Gothenburg, Sweden, who received curative external beam radiation therapy during the period 2006 to 2018. Patients previously treated with radical prostatectomy and those who received palliative radiation therapy were not included in the study. The study was approved by the Swedish Ethical Review Authority (registry no. 2019-03205).

### Collection and analysis of data

All available RTP CT imaging together with manually delineated target volumes and OARs of all included subjects were pseudonymized and uploaded to the research platform *www.recomia.org.*^21^ The platform automatically strips the image data of all identifying information during the upload process, except for the study code. The platform thereby hosts and processes fully deidentified image data only.

The first step of the study entailed a visual control of the existing manually delineated prostate and the surrounding OAR, that is, urinary bladder and rectum. This was done independently by 3 observers, including 1 urologist and 2 radiologists. Before the visual quality control, criteria of acceptance regarding organ delineations were developed in consensus during multidisciplinary meetings between the observers and experienced radiation oncology specialists. Acceptance criteria specified that the organ delineations should not overlap with a nearby structure and should cover the whole organ of interest. Delineations that did not meet the specified criteria were excluded from the study. Decisions were made on a per organ basis; for example, the delineation of the prostate of a patient could be excluded, whereas the urinary bladder of the same patient could be included in the study data set. In addition, the presence of contrast in the urinary bladder and the presence or absence of delineation of the seminal vesicles were noted. The seminal vesicles were delineated as part of the radiation target (prostate) for some of the patients, depending on the clinical stage of the PCa. Finally, the level of rectum contouring was noted, classifying the delineations as either below, at, or above the rectosigmoid junction. Patients with hip prostheses were excluded from the study because of streak artifacts.

**Figure 1:**
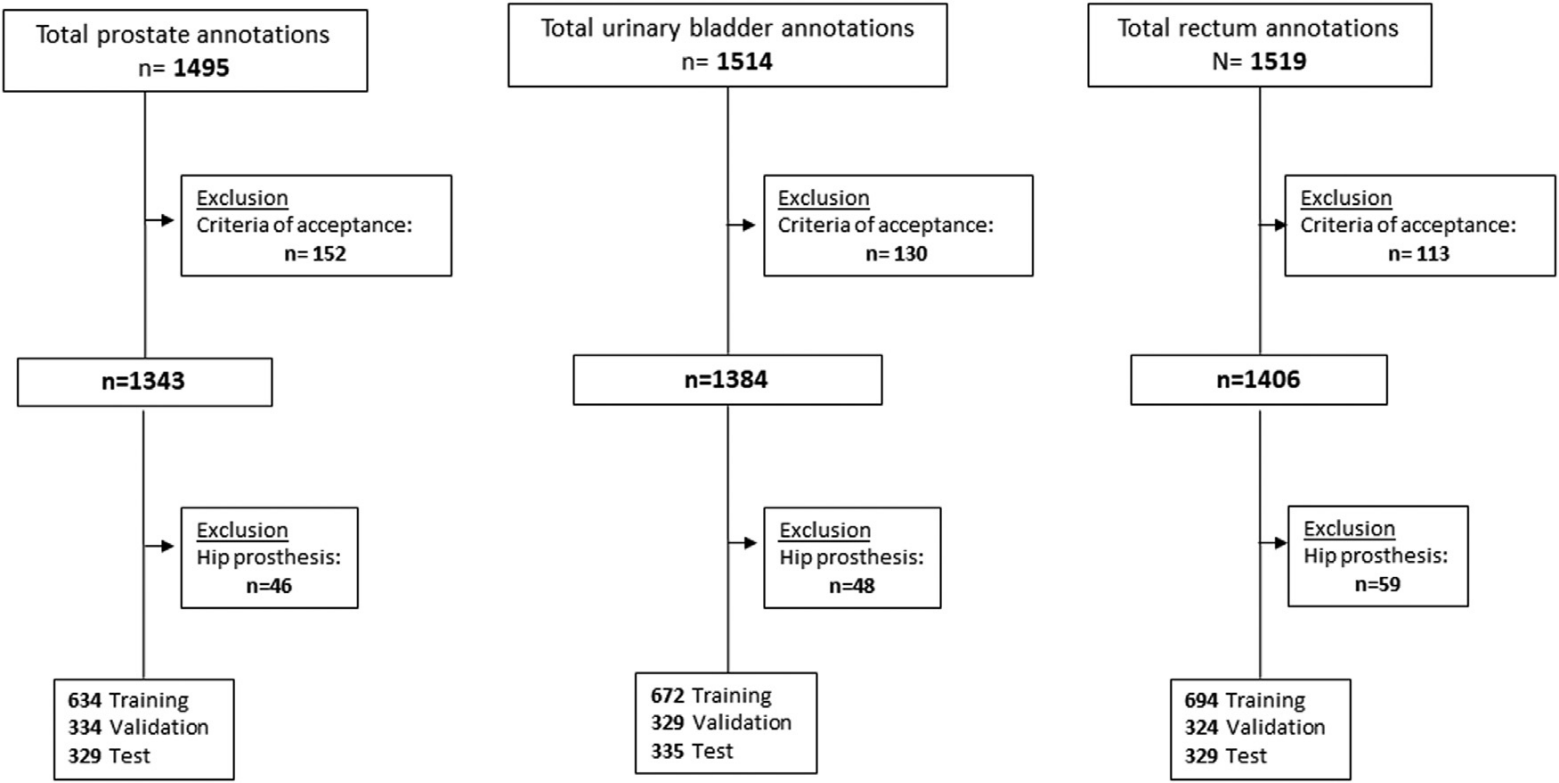
Flow chart showing the selection process of the study cohort.

**Figure 2:**
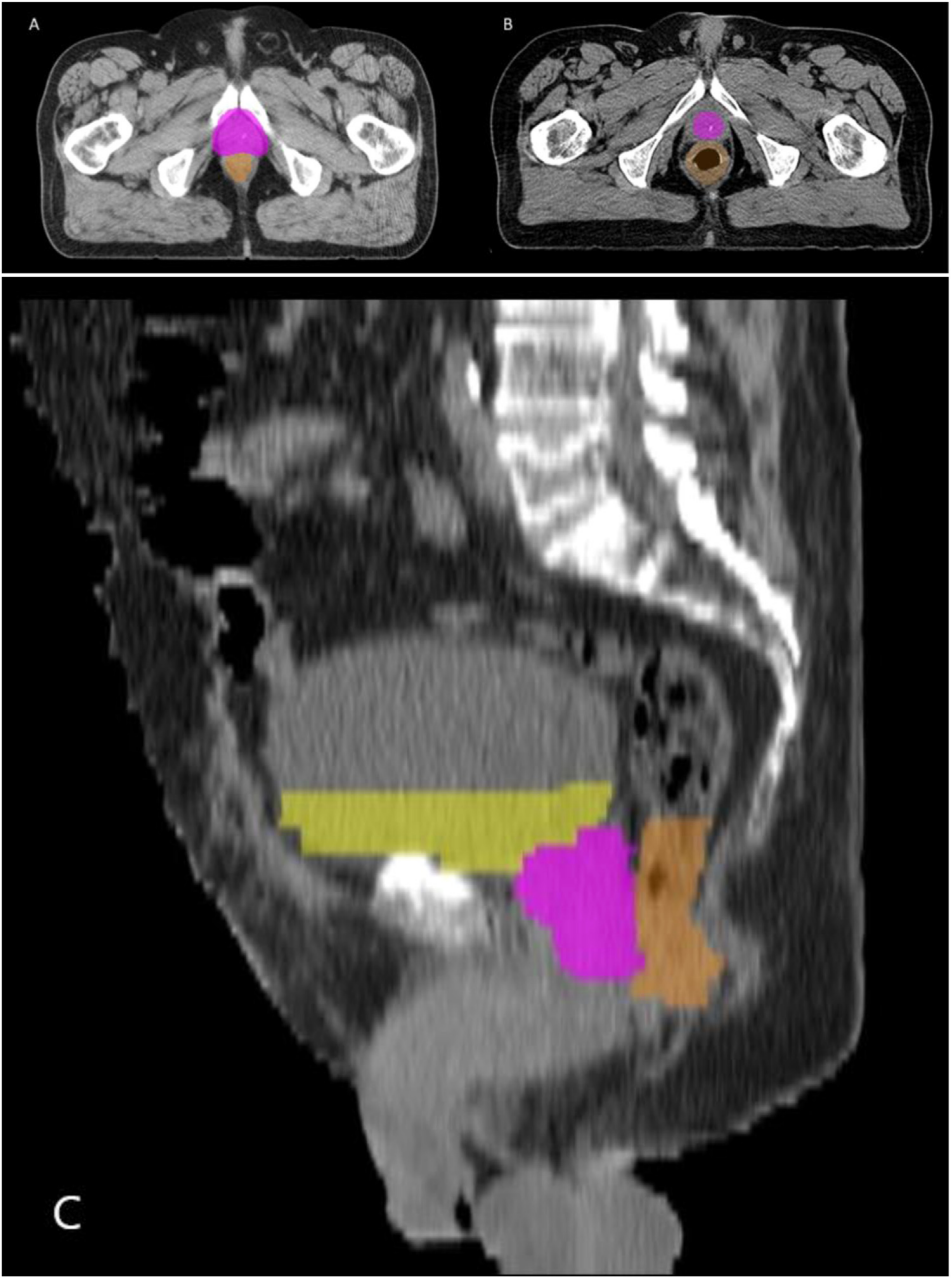
Pretreatment computed tomography scans of 3 representative patients with prostate cancer who did not meet the criteria of acceptance and were excluded from the training process. The colored delineations in yellow, pink, and orange correspond to manual contouring of the urinary bladder, prostate, and rectum, respectively. (A) and (B) Transverse plane of 2 patients from the initial cohort. The prostate delineation of patient A overlapped with nearby structures, especially anteriorly. The manual delineation of the prostate in patient B did not cover the whole organ. (C) Sagittal plane of a patient from the initial cohort. This case was excluded because large parts of the urinary bladder and rectum contouring were missing.

The included data set was then divided into a training set (approximately 50%), an internal validation set (approximately 25%), and a separate test set (approximately 25%). The internal validation set was used to fine tune the algorithm after training. The test data set was only used in the final validation after the AI algorithm had been finalized. The described procedure is shown in the flow chart of Fig. 1. Examples of the criteria for acceptance of delineations are shown in Fig. 2.

### Imaging

#### CT acquisition

All CT scans were obtained using a helical multidetector CT scanner (Toshiba Aquilion/LB or GE Medical Systems), using a scan field of view 400 to 700 mm, 120 kV, 500 to 1500 ms exposure time, and 55 to 500 mAs tube current modulation. The pelvic region was examined with the patients in the supine position. Administration of contrast media varied over time, depending on the local routines. The CT scans were reconstructed in the transverse plane with 1- to 5-mm slice thickness using filtered back projection. The convolution kernels used were FC07, FC13, FC17, and STD+.

#### AI algorithm

The algorithm was trained to automatically segment the prostate gland and the surrounding OAR of included patient CT images. The algorithm consisted of the fully convolutional neural network Unet-3D.^22^ The input images were rescaled to a pixel size of 1.0 × 1.0 × 3.0 mm (Digital Imaging and Communications in Medicine, DICOM, reference coordinate system) and clamped to (‒800, 800) Hounsfield units (HU).

#### Training protocol

Each epoch consisted of 20,000 training and 10,000 validation examples. Random background and foreground examples were selected such that each category was equally likely to be sampled. The input patches were augmented using rotations (‒0.15-0.15 radians), scaling (‒10% to 10%), and intensity shifts (‒100 to +100 HU). The Adam method^23^ with Nesterov momentum was used with an initial learning rate of 0.001. If there was no decrease in validation loss after 2 epochs, the learning rate was halved until a minimum of 0.00001 was reached. When there was no decrease in validation loss after 4 epochs, the training was stopped. After the initial training, the resulting algorithm was applied to all training images, and the sampling was updated with 20% of randomly selected misclassified pixels at least 3 mm from the foreground boundary (at most 20,000 pixels per image). This last step was repeated 4 times.

#### Loss

The algorithm was trained to classify each CT pixel as either background, prostate, seminal vesicle, urinary bladder, or rectum. Unfortunately, all of these structures were not annotated in all images, and sometimes the rectum was only partially annotated (stopping at a certain slice). To handle the partial labeling, a generalization of the categorical cross-entropy was used as a loss function. The algorithm was then trained to minimize this loss function. The loss function is described in the Appendix E1.

### Agreement between manual and AI-based measurements

All statistical analyses were done on the test data set, which was not used to train or design the algorithm. The agreement between the AI-generated image segmentations and manual delineations was evaluated with the DSC, mean surface distance (MSD), and Hausdorff distance (HD). DSC is a statistical tool commonly used in medical image segmentation to assess the agreement between 2 segmented volumes.^24^ The DSC ranges between 0 and 1, where 1 indicates perfect agreement. MSD and HD indicate the mean and the longest distance between 2 measurements, respectively.^25^

The segmentations of each organ were analyzed separately. For the prostate segmentations, those that included the seminal vesicles were analyzed separately from those that did not include the seminal vesicles. In cases where radiation oncologists did not differentiate between the prostate and seminal vesicles in manual segmentations, the AI analyzed the prostate and seminal vesicles together to generate a single segmentation. For the rectum segmentations, only those with an upper limit at the rectosigmoid junction were included in the analysis because that was the limit at which the AI was trained.

All image processing and the evaluation of AI performance were computed on the *www.recomia.org* research platform using Python programming language (version 3.9), and all statistical analysis was performed using SPSS Statistics 25 (IBM). The AI tool developed in this project is available upon reasonable request for research purposes at *www.recomia.org*.

## Results

In total, 1530 patients with RTP for PCa were included in the study. Out of the 1530 delineations, 152 prostate (10%), 130 urinary bladder (8.5%), and 113 rectum (7.4%) cases did not fulfil the criteria of acceptance and were excluded (Fig. 1). Further, all patients with uni- or bilateral hip prostheses were excluded. Examples of delineations that were excluded are shown in Fig. 2A,B. There was contrast in 861 urinary bladder cases, which did not cause any difficulties in the training of the algorithm and were all consequently included in the process.

**Figure 3:**
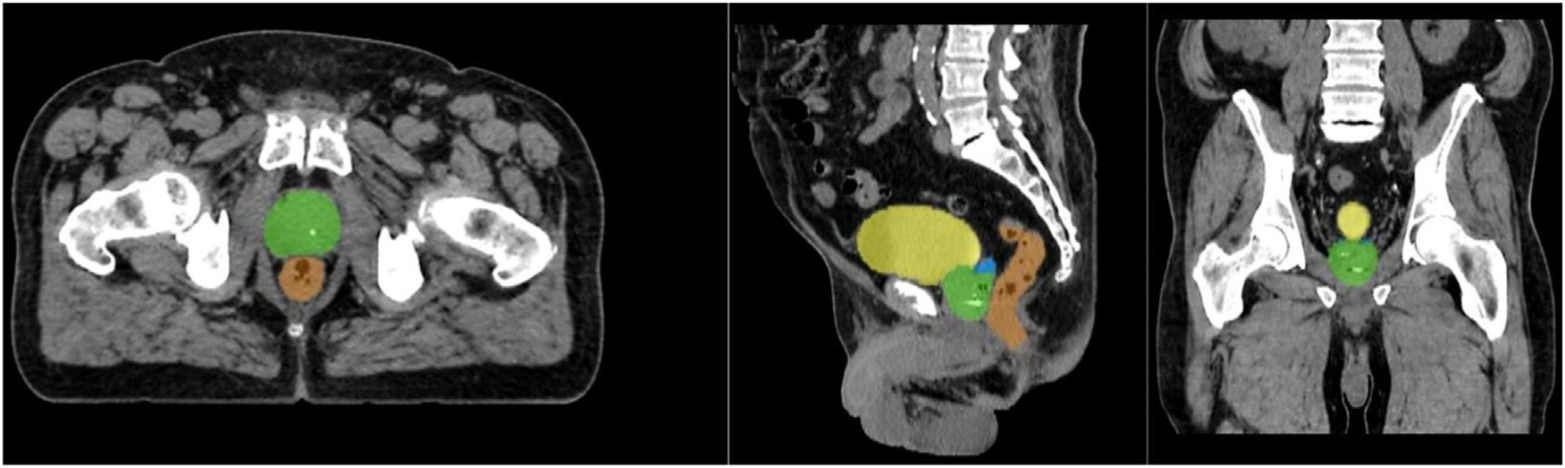
Example of organ delineations generated by the artificial intelligence algorithm in a computed tomography scan in axial, sagittal, and coronal planes in 1 of the patients of the final test group. The delineation of the prostate and seminal vesicles is shown in green and blue, and that of the urinary bladder and rectum is shown in yellow and orange, respectively. The Sørensen-Dice similarity coefficient between the artificial intelligence-based and the manual annotation of the prostate was 0.83, which is close to the median result of the study (0.77-0.86).

The results of the agreement between the manual and AI-generated segmentations in the test set are summarized in Table 1. The median DSC was 0.82 (IQR, 0.77-0.86) and 0.83 (IQR, 0.79-0.86) for the segmentations of 184 scans that were prostate only (56%) and of 145 scans of prostate with the seminal vesicles (44%), respectively. The corresponding median MSD was 1.7 (IQR, 1.3-2.3) and 1.6 mm (IQR, 1.4-2.2), respectively, and the median HD was 9.2 (IQR, 6.9-13) and 11.4 mm (IQR, 8.3-15.6), respectively. An example of a patient with segmentation agreements close to the median DSC is shown in Fig. 3.

**Table 1:** Comparison between the manual and AI-based organ delineations of the test set

	Prostate all n = 329			
Parameters	Prostate n = 184	Prostate and vesicles n = 145	Urinary bladder n = 335	Rectum n = 175
**DSC**				
median (IQR)	0.82 (0.77-0.86)	0.83 (0.79-0.86)	0.95 (0.92-0.96)	0.88 (0.86-0.9)
**MSD (mm)**				
median (IQR)	1.7 (1.3-2.3)	1.6 (1.4-2.2)	0.7 (0.6-0.9)	1.1 (0.8-1.5)
**HD (mm)**				
median (IQR)	9.2 (6.9-13)	11.4 (8.3-15.6)	6.7 (5.6-9.3)	13.5 (9.8-20.1)

*Abbreviations:* AI = artificial intelligence; DSC = Sørensen-Dice similarity coefficient; HD = Hausdorff distance; IQR = interquartile range; MSD = mean surface distance.

For all bladder delineations, there was good agreement between the 335 manual and AI-based segmentations, with a median DSC of 0.95 (IQR, 0.92-0.96); the median MSD was 0.7 mm (IQR, 0.6-0.9), and the median HD was 6.7 mm (IQR, 5.6-9.3). Out of these 335 cases, there were 174 CT scans with contrast and 161 CT scans without contrast in the urinary bladder. For the cases with contrast, the median DSC, MSD, and HD were 0.95 (IQR, 0.93-0.96), 0.7 mm (IQR, 0.6-0.9), and 6.9 mm (IQR, 5.6-9.6), respectively. For the cases without contrast, the corresponding values were 0.94 (IQR, 0.92-0.96), 0.7 mm (IQR, 0.6-0.9), and 6.6 mm (IQR, 5.6-8.9), respectively (Table 2).

**Table 2:** Results of the delineations in the test set according to contrast content in the urinary bladder

	Urinary bladder all n = 335		
Parameters	With contrast n = 174	Without contrast n = 161	
**DSC**			
median (IQR)	0.95 (0.93-0.96)	0.94 (0.92-0.96)	
**MSD (mm)**			
median (IQR)	0.7 (0.6-0.9)	0.7 (0.6-0.9)	
**HD (mm)**			
median (IQR)	6.9 (5.6-9.6)	6.6 (5.6-8.9)	

*Abbreviations:* DSC = Sørensen-Dice similarity coefficient; HD = Hausdorff distance; IQR = interquartile range; MSD = mean surface distance.

There were 175 rectum segmentations with an upper limit at the rectosigmoid junction that were analyzed, and the median DSC, MSD, and HD were 0.88 (IQR, 0.86-0.90), 1.1 (IQR, 0.8-1.5), and 13.5 mm (IQR, 9.8-20.1), respectively.

There were no cases where the AI completely failed to generate a segmentation for the target or OAR.

## Discussion

This study showed that automated delineations of the prostate and OAR can be successfully performed by our optimized algorithm for all analyzed pretreatment CT scans of patients with PCa. As expected, the performance of the algorithm was highly dependent on the reference standards. The AI-based contouring performed best on the urinary bladder, showing a DSC close to 1 and small differences in contour distance between automated and manual delineations. This is mainly because the boundaries of this structure are clearer than the prostate, despite the use of CT scans. The presence or absence of contrast in the bladder did not pose an obstacle to the automated segmentation.

The agreement between the algorithm and manual delineations of the prostate was good (DSC, 0.82). We previously reported the successful application of our algorithm on prostate positron emission tomography/CT imaging and obtained comparable measurements (DSC, 0.78) to those performed by experienced observers.^20^ In the current study, the improved training of the algorithm on a larger patient cohort provided more consistent delineations, with better agreement between the algorithm and the manual delineations of the prostate. Nevertheless, using manual delineations as ground truth did present some inherent challenges. Variations in manual delineations exist and appear to be multifactorial, with inter- and intraobserver variability as 2 of the prominent examples.^1^ Indeed, it has been shown that there is only 30% agreement among observers for the assessment of the prostatic base on CT scans.^26^ This was reflected in our cohort, where heterogeneity within the manual contouring of the prostate and seminal vesicles likely resulted in poorer agreement compared with the OAR. Anatomic variability across patients remains a challenge.^27^

The effect of heterogeneity among reference standards, and thus ground truth cases, was evident in the delineations of the rectum as well as the prostate. The pretreatment CT examinations were performed over a period of 12 years, during which the international guidelines concerning the delineation of seminal vesicles and the definition of an upper limit for rectum delineation changed. Collectively, these factors led to variability in manual organ contouring and consequently affected the training and performance of the AI algorithm. We included only cases with manual contouring of the rectum with an upper limit at the rectosigmoid junction to permit a direct comparison. The automated delineations of the rectum still performed well (DSC, 0.88), but the difference in HD was greater between the manual and AI-based delineations compared with the other organs of interest.

As CT remains one of the main imaging modalities in radiation therapy of PCa,^28^^,^^29^ studies on AI-based delineations on CT have direct applicability for clinical practice. Recently, Duan et al^30^ evaluated AI-based organ delineations on CT, reporting a DSC for the prostate (0.83) that was similar to that in our study. For the urinary bladder and rectum, the DSC values (0.93 and 0.85, respectively) were slightly lower than those in our study. However, this study comprised only 107 patients (84 as a training and validation set and 23 as a test set). Our study included a larger image data set, enabling better training of the algorithm.

The majority of reports to date have focused on AI algorithms for RTP on MRI.^14^^,^^31^^,^^32^ The advantages of MRI over CT for PCa RTP, mainly in the differentiation of the tumor inside the gland as well as in better soft-tissue contrast, are well known.^18^^,^^33^ Several recent studies have successfully applied AI algorithms on prostate MRI scans to achieve organ delineation with high degrees of agreement with manual approaches.^14^^,^^15^^,^^34^ However, interobserver variability still exists in MRI,^35^ and there is a particular concern near prostatic boundaries and seminal vesicles or surrounding organs, regardless of the choice of modality.^28^^,^^35^ Despite using CT scans only, our AI algorithm performed well and showed similar prostate contouring between manual and automated segmentations as well as successful delineations of the surrounding OAR with high DSC scores. This study demonstrated a DSC of 0.82 for prostate-only images, while Wang et al^14^ and Ushinsky et al^15^ used MRI for 90 and 299 patients and reported DSC values of 0.86 and 0.90, respectively. This small difference is expected, considering the better differentiation of the pelvic organs on MRI compared with CT.^36^

The main strengths of the study were the large data set used for training and testing and that the reference standards were segmented by experienced radiation oncologists with clinically relevant borders. To the best of our knowledge, there are few studies describing automated delineations of all relevant organs on pretreatment CT scans of patients with PCa. Other recent studies have shown satisfactory results after applying different algorithms on CT scans, including the pelvis.^37^^,^^38^ However, the study by Astaraki et al^37^ described the application of a semiautomated algorithm, whereas that by Chen et al^38^ included only 125 scans of the pelvis. Both studies were applied on different OARs, but not on the prostate. The algorithm of the current study was completely automated and applied to both the prostate and OAR. Additionally, a large patient cohort of 1530 patients with PCa was included, resulting in better training of the algorithm with good results.

One limitation of the current study was that it was carried out using CT scans only. This was partly because of the large number of CT scans available to enable sufficient training of the algorithm. For some patients included in the current study, MRI scans had been obtained for more detailed anatomic imaging, thus providing additional data to radiation oncologists. Reference standards with manual contouring using both CT and MRI data were accordingly more accurately delineated and could have positively affected the training of the algorithm. Future studies using a combination of CT and MRI could potentially increase the accuracy of the algorithm.

Another limitation was that the patient cohort of the study was rather heterogeneous. This reflects the real-world clinical setting upon which the manual delineation set was based. As noted, there were variations in manual delineations because patients were examined over a 12-year period during which the guidelines for RTP changed. In the manual delineations of the rectum, the upper limit for contouring varied over time. Further, the seminal vesicles of the cohort were manually delineated depending on the stage of PCa, as traditionally, seminal vesicles were included in the RTP in patients with high-risk PCa but not in those with low or intermediate risk. This had an effect on the performance of the algorithm because its training was highly dependent on manual contouring. Patients with hip prostheses were excluded from the analysis because of major artifacts in the pelvic region; however, these hampered the manual annotations as well. Finally, the agreement measures (DSC, MSD, and HD) of this study are purely mathematical in nature, which may ignore segmentation errors that would be clinically unacceptable.

Qualitative evaluations of the algorithm's performance are needed before clinical applications can be made available. An upcoming visual grading study is planned.

## Conclusion

Fully automated AI-based delineations of the treatment target organ and OAR in PCa RTP based on CT scans are feasible and were comparable to manual segmentation in the majority of cases in the study. This could lead to large resource savings, greater standardization, and improved outcomes for patients with PCa.

## Disclosures

The authors have no conflicts of interest to declare.

## Appendix Supplementary materials

Supplementary material.pdf Image, application 1

## Footnotes

Supplementary material associated with this article can be found in the online version at doi:10.1016/j.adro.2023.101383.
