# Supplementary material for: Artificial Intelligence-Based Organ Delineation for Radiation Treatment Planning of Prostate Cancer on Computed Tomography
Source: Adv Radiat Oncol. 2023 Oct 14;9(3):101383. doi: 10.1016/j.adro.2023.101383 (PMC10943520; doi:10.1016/j.adro.2023.101383)
Supplement: Supplementary material.pdf [file mmc1.pdf]

Supplementary material

Loss function

The goal of the algorithm is to classify each CT pixel as either background, prostate, seminal vesicle, urinary bladder, or rectum. Unfortunately, all these structures are not annotated in all images, and sometimes the rectum is only partially annotated (stopping at a certain slice). To handle this, we considered each pixel, i, as annotated with a label set, S_i, consisting of one or multiple labels. The loss for this pixel, L_i, is computed as follows:

$$L_i = -\log \sum_{k \in S_i} y_k,$$

This is a generalization of the categorical cross-entropy.

For example, if the bladder and rectum are not annotated in a specific image, we no longer know if an unlabeled pixel is the background, bladder, or rectum. Hence, we give all unlabeled pixels the label set:

$$S_i = \{background, bladder, rectum\}$$

The loss for such a pixel, if the network outputs are $y_{background} = 0.2$, $y_{bladder} = 0.7$, and $y_{rectum} = 0.1$, will be:

$$-\log(0.2 + 0.7 + 0.1) = 0$$

Similarly, in an image where the seminal vesicles are segmented along with the prostate, any such pixel will get the label set:

$$S_i = \{prostate, seminal\ vesicle\}$$

and if the network outputs $y_{prostate} = 0.3$, $y_{vesicle} = 0.6$, $y_{background} = 0.1$, then the loss will be:

$$-\log(0.3 + 0.6) \approx 0.046.$$

The same idea is used when the rectum is only partially annotated. Any unlabeled pixels above the last slice where rectum is annotated are given the label set:

$$S_i = \{background, rectum\}$$

Below the level that the rectum is not annotated, we do not have this uncertainty.
